# Prosody matters: Preserved prominence marking strategies in people with Parkinson’s disease independent of motor status

**DOI:** 10.1371/journal.pone.0308655

**Published:** 2024-08-20

**Authors:** Tabea Thies, Michael T. Barbe, Doris Mücke

**Affiliations:** 1 Department of Neurology, Faculty of Medicine and University Hospital Cologne, University of Cologne, Cologne, Germany; 2 IfL Phonetics, Faculty of Arts and Humanities, University of Cologne, Cologne, Germany; University of Missouri Columbia, UNITED STATES OF AMERICA

## Abstract

While many studies focus on segmental variation in Parkinsonian speech, little is known about prosodic modulations reflecting the ability to adapt to communicative demands in people with Parkinson’s disease (PwPD). This type of prosodic modulation is important for social interaction, and it involves modifications in speech melody (intonational level) and articulation of consonants and vowels (segmental level). The present study investigates phonetic cues of prosodic modulations with respect to different focus structures in mild dysarthric PwPD as a function of levodopa. Acoustic and kinematic speech parameters of 25 PwPD were assessed in two motor conditions. Speech production data from PwPD were collected before (medication-OFF) and after levodopa intake (medication-ON) by means of 3-D electromagnetic articulography. On the acoustic level, intensity, pitch, and syllable durations were analyzed. On the kinematic level, movement duration and amplitude were investigated. Spatio-temporal modulations of speech parameters were examined and compared across three different prosodic focus structures (out-of-focus, broad focus, contrastive focus) to display varying speech demands. Overall, levodopa had beneficial effects on motor performance, speech loudness, and pitch modulation. Acoustic syllable durations and kinematic movement durations did not change, revealing no systematic effects of motor status on the temporal domain. In contrast, there were spatial modulations of the oral articulators: tongue tip movements were smaller and lower lip movements were larger in amplitude under levodopa, reflecting a more agile and efficient articulatory movement under levodopa. Thus, respiratory-phonatory functions and consonant production improved, while syllable duration and tongue body kinematics did not change. Interestingly, prominence marking strategies were comparable between the medication conditions under investigation, and in fact, appear to be preserved in mild dysarthric PwPD.

## Introduction

Speech production is adjusted for producing prosodic prominence in order to highlight specific parts of an utterance and to indicate information structure along a continuum of non-prominent to prominent utterance constituents, e.g. words or larger focus domains [1, 2]. Thus, prosody with its linguistic function is an essential part of daily life communication. Moreover, conveying prosodic prominence is a complex process that requires fine-tuned adaption of speech motor control in regards to its communicative demands, as all speech systems are involved to varying degrees [3]. Multiple phonetic cues, such as variation in fundamental frequency (speech melody, perceived pitch [2, 4–6]), intensity (perceived loudness [7–9]), and articulatory features of consonant and vowel production [10–13] are described to encode prosodic prominence in connected speech. One specific area in which prosodic cues are modulated is to indicate information structure in terms of focus marking [1]. Focus marking guides the listeners’ attention to important or new information by highlighting specific parts of utterances within communicative contexts, while already given or generally known information is classified as less important and is not specifically highlighted [2]. Given information is considered out-of-focus and moved to the non-prominent “background” position. In the question-answer-pair in (1), the question triggers the focus structure in the answer: The target word “Lena” is in the background, while “the brother” is highlighted by receiving the nuclear pitch accent.

(1)

 Q: Was the grandpa waving to Lena?

 A: [The brother]_F_ [was waving to Lena]_B_.

Prominent information in focus condition can be produced in “broad focus” or “contrastive focus” condition. Given the question-answer-examples below, the whole answer in example (2) is in broad focus condition assuming that the information is new to the listener (all-new sentence). In example (3) only the girl name “Lena” is focused and expresses the correction for the alternative name given in the question while the rest of the utterance is in background position.

(2)

 Q: What happened?

 A: [The grandpa was waving to Lena]_F_.

(3)

 Q: Was the grandpa waving to Nora?

 A: [The grandpa was waving to]_B_ [Lena]_F_.

Prosodic prominence in terms of multiple phonetic adjustments increase from background to broad focus and further to contrastive focus condition [14, 15]. For German, pitch is the strongest correlate of prosodic prominence and reflected in terms of pitch accents that are placed on stressed syllables in focus condition [3]. Particularly (steep) rising pitch movements are expected to signal prominence [2, 6]. Note that pitch movements of bitonal pitch accents often start before and end after the stressed syllable. Further, the intensity level should increase, especially comparing out-of-focus and focused syllables related to unaccented and accented syllables [3]. Lastly, articulation is more distinct and acoustic segments are prolonged by means of the sonority expansion strategy and the hyper-articulation to enhance paradigmatic and syntagmatic contrast between focused and out-of-focused elements [9, 10, 12, 16].

The process of highlighting focused elements requires variation in the amount of vocal effort in order to serve as a function of prosodic structure [4], which can be related to the model of hyper- and hypospeech (H&H Model) [16]: In non-prominent positions, the physical control system tends to minimize the amount of speech effort for the involved subsystems (hypo-articulation, low-cost behaviour of the speech system), while an increase in effort lends prominence to units of speech (hyper-articulation, high-cost behaviour) [16].

People with Parkinson’s disease (PwPD) develop a motor speech disorder (hypokinetic dysarthria) that leads to a hypo-functioning of the speech motor system. Particularly, pitch modulation, loudness variation, voice quality, imprecise articulation, speaking rate, and pausing behavior are affected by this type of dysarthria [17]. The terms dysprosody, monopitch, and monoloudness are often reported in the clinical context of PD speech and suggest that prosodic prominence marking could be affected in PD, especially on the intonational level. These difficulties should only become apparent in connected speech such as prosodic phrases or utterances [18, 19], while they should not be detectible in more artificial speech tasks, such as fast syllable repetitions or sustained vowel production. It has been shown that PwPD were able to increase speech effort to highlight important words in an utterance [20, 21], but the degree of modulation to signal prosodic prominence differed from neurotypical speakers [20–24], especially regarding the production of pitch accents that were found to have a narrower pitch range [20, 24] and earlier pitch peak attainment [21] in PwPD compared to healthy controls affecting prosodic categories and phrasing [22]. Hertrich and Ackermann (1993) also reported on decreased durational contrasts in PwPD, while pitch accent types were preserved. Most studies investigated prosodic feature adjustment on the acoustic level, while only little research considered the kinematic observation of the underlying articulation containing overlapping speech movements of lips, tongue, and jaw. It has been shown that articulatory movements of the jaw, the lips and the tongue increase in duration and amplitude to encode prosody, such as stress or focus structure [25–28]. Thus, prosodic functions are potentially preserved on the kinematic level too.

(Speech) motor deficits in PD are caused by a progressive loss of dopaminergic cells in the substantia nigra. While levodopa is proven to be an effective treatment for improving gross motor control in PD [29], the effect on motor speech and the prosodic speech system is under debate. Previous acoustic studies report variable findings that include either changes of prosodic speech parameters due to levodopa intake, such as increased intensity [30–32], better pitch control [22, 31], shorter syllable durations as indicator of faster articulation rate [33], or no change at all regarding pitch [30, 33–37], articulation/speech rate [30, 32, 34, 38], and intensity [33]. With regard to vowel space changes, smaller vowel spaces [39] but also larger vowel spaces [40] were observed reflecting contradictory results. Changes seem to be speaker-specific as changes occurred only in a subgroup of patients [41]. The number of kinematic studies is far lower than the number of acoustic studies. To date, results on the kinematic level reveal that speech kinematics respond to levodopa. For the lip system, faster [42, 43] and smoother movements [43] as well as greater perioral stiffness and larger movements [44] were observed, leading to a higher degree of acoustic energy radiating from the mouth. For the tongue system, shorter, larger, and faster tongue body movements were determined under levodopa [32], reflecting a higher degree of efficiency in articulatory behavior. So far, kinematic studies that particularly investigate *consonant* and *vowel* production on a sub-syllabic level with respect to the involved articulators of the oral system are lacking.

To explain variable results, recent studies highlight that the responsiveness to levodopa of speech parameters, particularly voice features and dysfluencies, in PwPD differ dependent on the speech parameter severity in a condition when levodopa was withdrawn [45, 46]. This indicates that the likelihood that speech function will improve dramatically with levodopa is increased in PwPD with poor speech function in medication OFF condition. Another domain that could be considered to explain variable results is the instruction that was given in previous experiments on how to perform the speech task. For example, in the studies of Tykalova et al. [20] or Gaviria [24], PwPD were explicitly instructed to focus certain words. In contrast, a question-answer-scenario as used by Thies et al. [21] and Frota et al. [22] explores prosodic prominence in terms of focus marking in a rather naturalistic speech design. Different instructions exhibit different forms of speech motor processes that might provoke different prominence marking strategies in PwPD regardless of the overall study design.

To summarize, previous studies report inconclusive results to what extent levodopa affects prosodic speech parameters. The interplay between intonational and articulatory modifications of prosodic prominence marking of natural sentence productions in PD is still unclear. Thus, this experiment is designed to examine how PwPD modulate prosodic speech parameters under varying speech demands under three focus structures (background, broad focus, and contrastive focus), and whether their prosodic adjustments change due to the medication state (ON and OFF) as well as the corresponding motor condition. Note, that focus structure is tightly connected to prosodic prominence, revealing a systematic increase of vocal effort from background to broad focus to contrastive focus in many phonetic parameters in neurotypical sentence productions in German as well as pitch accent placements on focused elements [3]. In our study, we use a levodopa challenge test to investigate medication effects on motor speech in PD. We examine speech not only on the acoustic level (pitch, intensity, syllable duration) but also on the kinematic level to gain insights into movement patterns (duration, amplitude) of the lower lip, tongue tip and tongue body. We hypothesize that PwPD can signal prosodic prominence on the acoustic and kinematic level by using the same strategies as reported for healthy controls. While the effect of levodopa on acoustic parameters remains unclear, faster and larger kinematic movements are expected under medication.

## Methods

### Ethics

The study was approved by the local ethics committee of the University Hospital of Cologne (protocol code: 18–425; date of approval: 8 February 2019). Written informed consent was obtained from all subjects prior to study participation. Participants were recruited in the period from 07.05.2019 to 10.06.2021.

### Participants

25 individuals that have been diagnosed with PD prior to study inclusion according to the UK brain bank criteria with idiopathic PD were included in the study [47]. Participants were native speakers of German and had mild to moderate dysarthric symptoms according to a screening of a speech therapist with expertise in neurogenic speech disorders working in our clinic. The speech screening further excluded the presence of other speech and language problems, such as aphasia, apraxia of speech, or developmental speech disorders. Speech was screened based on the PwPDs’ performance in several tasks under regular medication dosage: maximum vowel phonation, oral diadochokinesis, reading, spontaneous speech, and modulation of loudness and pitch. The dysarthria severity was based on the conglomerated speech performance in all tasks considering fluency, speech tempo, articulatory precision, prosody, voice quality, speech loudness and the coordination of speech and breathing. Reduced articulatory precision, breathy voice quality, reduced prosody and deviance in speech tempo (faster or slower) were main domains of speech impairment in this cohort (Table 1). Speech severity was rated under regular medication condition so that the speech therapist was not blinded to the participants motor status. Furthermore, speech samples recorded within the experimental session (single sentences in medication-OFF condition) per speaker were randomly presented to naive listeners. Each speech sample was rated by 42 naive listeners on a visual analogue scale, ranging from 1 (unintelligible) to 101 (intelligible), to assess speech intelligibility. Higher values represent better intelligibility (Table 1). The inter-rater reliability was high, as the average ICC was .92 with a 95% confidence interval from .85 to .97 [F(15,672) = 12.4, p < .001]. Naive listeners were recruited via Prolific (www.prolific.com) and the visual analogue scale was presented in SoSci Survey [48]. Intelligibility scores for two speakers are unavailable because the gain levels during their recordings were not controlled, making their intensity levels incomparable to the other audio signals.

**Table 1:** Participant characteristics and results of neuropsychometric assessments. LEDD = Levodopa Equivalent Daily Dose, MMSE = Mini-mental state examination (cut-off < 19 [55]), PANDA = Parkinson Neuropsychometric Dementia Assessment (cut-off < 14 [56]), BDI = Beck-Depression-Inventory-II (cut-off > 13 [57]).

PD	sex	Age (years)	Disease duration (years)	LEDD (mg)	Levodopa response (%)	Speech impairment (regular med-ON)	Intelligibility score (med-OFF)	MMSE	PANDA	BDI
PD01	m	69	13	983	-65	moderate	71	24	23	0
PD02	f	51	6	1099	-48	mild	86	30	30	6
PD03	m	61	19	1501	-70	moderate	-	29	25	9
PD05	m	54	5	1030	-73	mild	61	29	22	6
PD06	m	63	6	837	-42	moderate	53	29	25	1
PD07	f	56	6	726	-44	mild	87	30	27	0
PD08	m	68	12	1224	-85	mild	40	29	24	3
PD10	f	70	20	1452	-68	mild	74	28	14	9
PD11	m	62	8	1267	-48	mild	77	28	28	9
PD12	m	58	2	901	-45	mild	-	30	26	7
PD13	m	53	4	540	-52	mild	73	29	27	5
PD14	f	69	13	1254	-48	mild	59	29	29	10
PD16	m	56	1	300	-50	mild	48	29	27	3
PD19	m	60	8	1728	-65	mild	83	29	25	10
PD20	m	42	7	745	-44	mild	51	30	24	3
PD21	m	59	4	1050	-70	mild	90	30	20	4
PD22	m	56	7	1105	-36	mild	69	27	14	12
PD24	m	66	11	700	-42	mild	67	28	19	1
Mean (sd)	14 m, 4 f	60 (7)	8 (5)	1025 (346)	-55 (14)	15 mild, 3 moderate	68 (26)	29 (1)	24 (5)	5 (4)

All participants were recruited during an in-hospital stay and were tested by means of a levodopa challenge test. This test examines the effect of a standardized levodopa dosage on motor functions by comparing medication-OFF and medication-ON condition [49, 50]. To achieve the OFF condition, PD medication was withdrawn for at least 12 hours as all participants were undergoing levodopa therapy regularly. For the ON condition, each patient received a predetermined oral dose of 200 mg soluble levodopa (2 x 100/25 mg levodopa/benserazid tablets) in accordance with our clinical standard. However, baseline levels of levodopa equivalent daily doses (LEDD) were documented (Table 1) [51].

Part III of the ‘Unified Parkinson’s disease ratings scale’ [52] was used to monitor motor functions of all participants in both medication conditions and to calculate the levodopa response [53]. Afterwards, participants were divided into levodopa responders and non-responders based on the percentage difference in the UPDRS III values between both medication conditions [54]. As suggested in the guidelines of the German Society for Neurology, participants that had a percentage change of UPDRS III values from OFF to ON condition below 30% were considered non-responders and were therefore excluded from the final analysis [49]. Note, that Tykalova et al. used a cut-off of 20% to investigate short-term effects of levodopa on speech [54].

Furthermore, all participants needed to pass a screening protocol to exclude the presence of dementia or depression (Table 2). Accordingly, seven PwPD had to be excluded from the analysis after the study inclusion; three because of their results of the neuropsychometric assessments, and four because they were non-responders. The final data set consists of 18 PwPD, and their characteristics are reported in Table 1.

**Table 2:** Examples of question-answer pairs to elicit different focus structure. Original German sentences on the left, translated sentenced in English on the right. Table is adapted from [58].

**(i) Background** (girl’s name is already given, not accented)	
Hat der Opa der Luna gewunken?	(Was grandpa waving to Luna?)
Der Bruder hat der Luna gewunken.	(The brother was waving to Luna.)
**(ii) Broad focus** (girl’s name is new, accented)	
Was ist passiert?	(What happened?)
Der Opa hat der Luna gewunken.	(The grandpa was waving to Luna.)
**(iii) Contrastive focus** (girl’s name is new and name corrected, accented)	
Hat die Schwester der Nora gewunken?	(Was the sister waving to Nora?)
Die Schwester hat der Luna gewunken.	(The sister was waving to Luna.)

### Speech data elicitation

#### Experimental set-up

Speech and motor data were assessed in the OFF condition first, and second in the ON condition, 30 to 40 minutes after intake of soluble levodopa. The motor assessment preceded the speech recordings in each medication condition. Acoustic and kinematic speech data were recorded with an electromagnetic articulograph (AG 501, Carstens Medizinelektronik GmbH). Small sensors were attached to the lower lip, tongue tip, and tongue body by using tissue adhesive to track articulatory movements. Tongue sensors were placed approximately 1 cm (tongue tip) and 4 cm (tongue body) from the tip of the tongue. Additionally, two sensors were attached behind the ears, which functioned as reference sensors for head-correction. The raw data were converted into positional data first and then the head movement was corrected and rotated into a head-based coordinate system using a biteplane recording and the respective software provided by Carstens. Acoustic data was recorded by using a condenser microphone headset (AKG C 544 L, 44.1 kHz/16 bit) to keep a constant distance of 7 cm from the mouth to the microphone. The gain level was adjusted between recording session and conditions. Therefore, a pure tone was recorded as a first stimulus, which was then used as a reference tone to extract corrected intensity values later on.

#### Speech task

Participants were placed in front of a monitor that presented a programmed game-like application, consisting of a question-answer-scenario to elicit more natural speech. By varying the displayed scenario, the context, and the question, participants were instructed to produce the target sentences in different focus categories. Thus, target words were produced in three different focus categories: background position, broad focus, and contrastive focus. Ten disyllabic girl names (C_1_V_1_.C_2_V_2_-structure) with word stress on the first syllable were chosen as target words (i.e. Lina, Mila, Lena, Mela, Lani, Mali, Loni, Moli, Mula, Luna) that were embedded in a predefined sentence structure. The first consonant C_1_ was either a bilabial nasal [m] to track lower lip movements, or a lateral [l] to track the tongue tip movement. The vowel V_1_ was one of five German corner vowels [i, e, a, o, u] to investigate the movements of the tongue body within the whole articulation space. The question-answer pairs per focus categories are exemplified in Table 2, along with a detailed description and explanation of the speech material [58].

In all examples (Table 2), the focus structure of the answer containing the target word is triggered by the type of question [3, 4, 59]. In the answer of the (i) background condition, the subject “the brother” is in focus, while “was waving to Luna” is out-of-focus in background condition. We can expect that the target word “Luna” will not receive a pitch accent in this condition (unaccented) and will therefore receive no prominence. In (iii) contrastive focus condition, the target word “Luna” is in focus, while the “the sister was waving to” is in the background condition. We can expect that the target word will receive a pitch accent (accented) and will be produced with a high amount of prominence. Background versus contrastive focus are the most diverging focus structures. In (ii) broad focus, we can expect that the entire sentence “Die Schwester hat der Luna gewunken” will be in focus, reflecting an “all-new” sentence. The target word “Luna” is the last noun in the sentence and will receive the nuclear pitch accent (accented), but we can expect an intermediate level of prominence on the target word (background<broad focus<contrastive focus).

Furthermore, the task tests the ability and the flexibility of the speech system in PD and whether it is limited by the motor status (OFF vs ON). The elicitation of background productions was included to investigate target word productions in non-prominent positions to analyze whether PwPD can actively reduce articulatory effort. The contrastive focus category was included to determine if they can increase functional load by strongly modulating articulatory parameters. The distinction between background and broad/contrastive focus is considered a comparison across accentuation, while the distinction between broad and contrastive focus is considered as comparison within accentuation. For prominence, an increase in intensity values and durations, adjusted pitch modulation in terms of (steeper) rising pitch contours as well as more distinct articulation is expected [3, 4, 9, 11].

Participants did not receive any instruction to specifically focus the target words but were asked to answer the question in the given sentence structure and to speak as if they were talking to a real person. A test phase was included in which all target words were produced in isolation and three test trials were carried out to provide participants with the opportunity to familiarize themselves with the experimental setting.

### Data processing and measures

**Fig 1:**
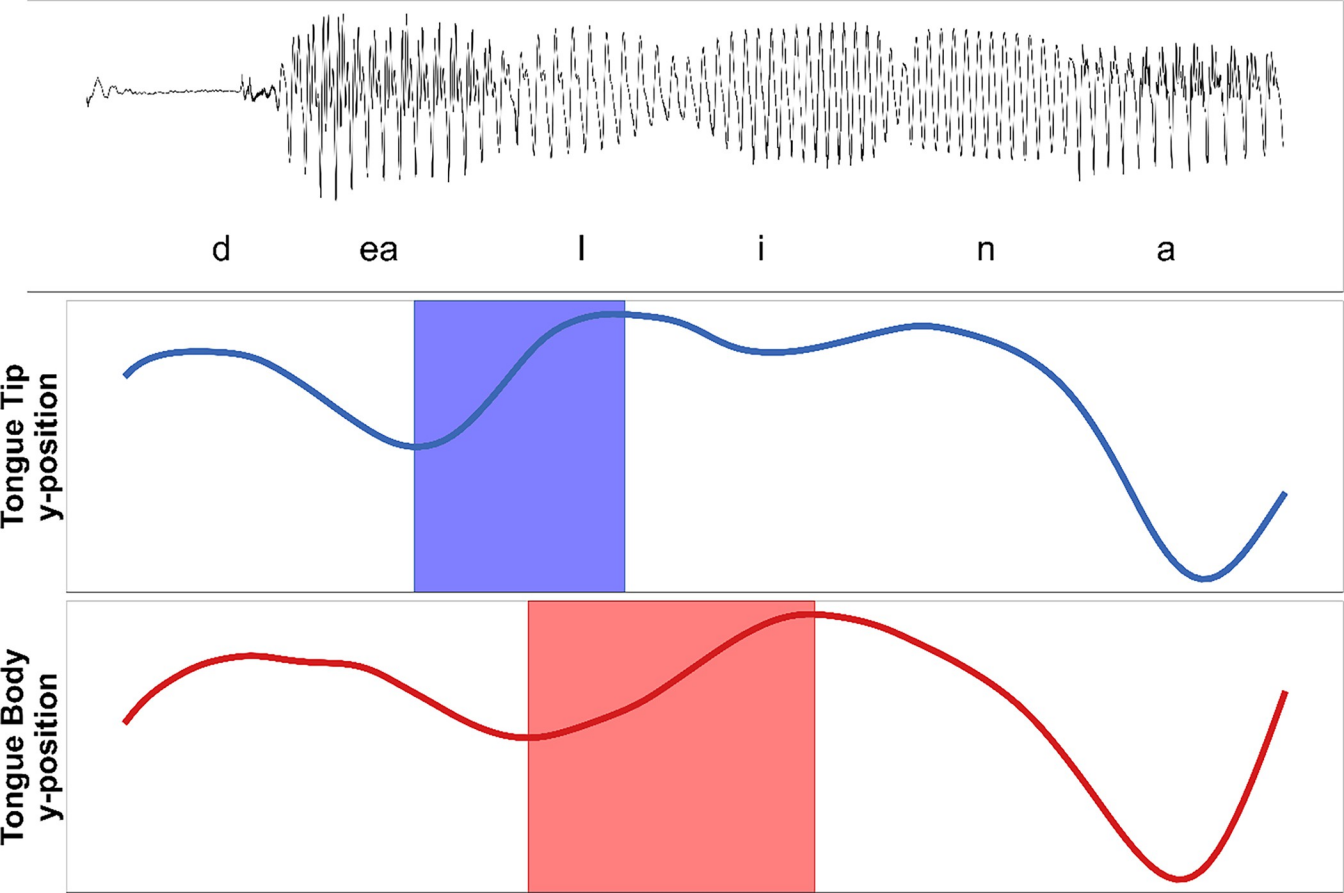
Acoustic and kinematic signals for the utterance /der lina/. Acoustic waveform at the top, movement trajectories of the tongue tip and the tongue body below. The highlighted areas frame the interval from the start to the target of each movement. The tongue tip (in blue) is raised for the consonant /l/, and the tongue body (in red) is raised for the vowel /i/.

Parameter adjustments are analyzed on prosodic features that are locally related to the first syllable of the target words. On the acoustic level, target words, stressed C_1_V_1_-syllables, and their respective segments were annotated according to the speech waveform and the wide-band spectrogram. The analysis on the kinematic level focuses on the vertical dimension (raising and lowering of the articulator). Two landmarks were defined for each articulatory movement by means of zero-crossings in the respective velocity trace for each articulatory movement: i) start of the movement and ii) target of the movement (Fig 1). Data was manually annotated in the EMU-webAPP of the EMU-SDMS environment [60]. The following parameters were extracted and calculated by means of the “emuR” [61] and “praatR” [62] packages in the software R.

### Acoustic measures

*Pitch range (st)*. The pitch range is a spatial measure of F0 modulation and related to the frequency difference of the pitch movement within the first syllable from its start to its end.

*Intensity (dB)*. The mean intensity (perceived loudness) of the vocalic segment V_1_ was computed.

*Acoustic syllable duration (ms)*. The temporal interval between the start of the consonant C_1_ and the end of the vowel V_1_.

### Kinematic measures

*Movement duration (ms)*. The temporal interval between the onset and the maximum target indicating how much time the movement takes was calculated for each articulator separately (tongue tip, tongue body, and lower lip).

*Movement amplitude (mm)*. The relative positional difference between the onset and the maximum target on the vertical axis, indicating the spatial distance that has been traveled by the articulator. This parameter was calculated for each articulator separately (tongue tip, tongue body, and lower lip).

### Statistical analysis

Data exploration and statistical analyses were conducted by using the software R (version 4.2.2; R Core Team, 2023). The statistical analysis of acoustic and articulatory speech outcomes was performed with the “lme4 package” [63]. In total, 1080 productions were recorded (18 participants x 2 medication conditions x 3 focus conditions x 10 words). However, only 1024 productions went into analysis of which 509 were produced by PwPD in the OFF and 515 in the ON condition, as some productions were excluded due to mispronunciation or sensor tracking errors. Linear mixed models were built with treatment condition and focus category as predictor variables, both coded as factor in the model. Random intercepts were included for speaker and target words. The raw data was inserted in the models and no data manipulation in the form of a transformation was used. Main effects were validated by comparing the test model (with the critical predictor) to a reduced model (without the critical predictor) via likelihood-ratio tests. P-values are based on these comparisons and post-hoc analyses (Tukey method) were completed on the full model by using the “emmeans package” [64] if the main effect of the critical predictor or the interaction term between both fixed effects were found significant. Nine models were run in total (pitch range, intensity, syllable duration, 3 x movement duration, 3 x movement amplitude).

## Results

### Acoustics

In the following section, the acoustic results on pitch range, intensity, and duration of the target syllable are presented. Means and standard deviations for the measures are displayed in Table 3. Data points in the figures represent the raw data. Each dot represents data of one participant averaged across target words/ vowels.

**Table 3:** Means and standard deviations of acoustic parameters per medication condition and focus category.

	**Pitch Range (st)**		
	background	broad	contrastive
OFF	1.9 (2.2)	2.7 (2.0)	3.9 (3.3)
ON	1.7 (1.6)	3.3 (2.4)	4.2 (2.8)
	**Intensity (dB)**		
	background	broad	contrastive
OFF	71.7 (4.5)	73.5 (4.9)	74.3 (5.0)
ON	75.1 (5.9)	77.3 (6.4)	77.6 (6.1)
	**Syllable duration (ms)**		
	background	broad	contrastive
OFF	202 (50)	217 (51)	224 (55)
ON	200 (44)	218 (53)	229 (55)

### Pitch range

**Fig 2:**
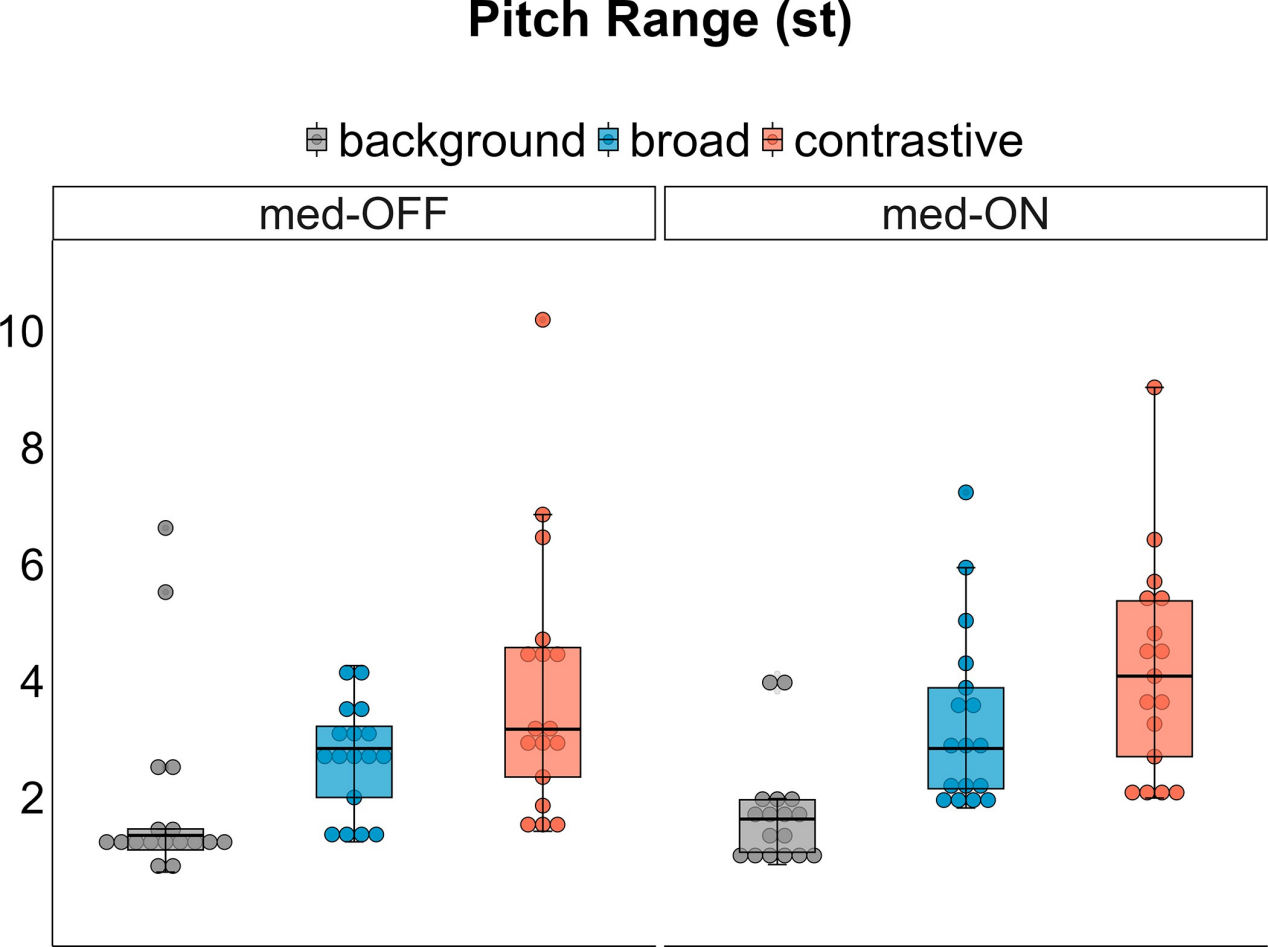
Pitch movement range within the first syllable of the target words per medication condition and focus category.

There is a main effect of medication condition [X^2^(2) = 4.2383, p = .040] and of focus category [X^2^ (2) = 166.14, p < .001] on pitch range. Post-hoc comparisons reveal that the pitch range is smaller in the OFF compared to the ON condition (mean difference = -0.277 st, p = .039) and also differs between each focus category (background vs. broad: mean difference = 1.21 st, p < .001 | background vs. contrastive: mean difference = 2.22 st, p < .001 | broad vs. contrastive: mean difference = 1.00 st, p < .001). The condition x focus interaction was also significant [X^2^(2) = 6.9319, p = .031], indicating that the pitch range in the background and contrastive focus categories does not differ between the medication conditions. However, the pitch range is lower in broad focus in the OFF condition compared to the ON condition (mean difference = 0.669 st, p = .046, Fig 2), while there is a significant difference within broad and contrastive focus (mean difference = 0.845 st, p = .004).

### Intensity

**Fig 3:**
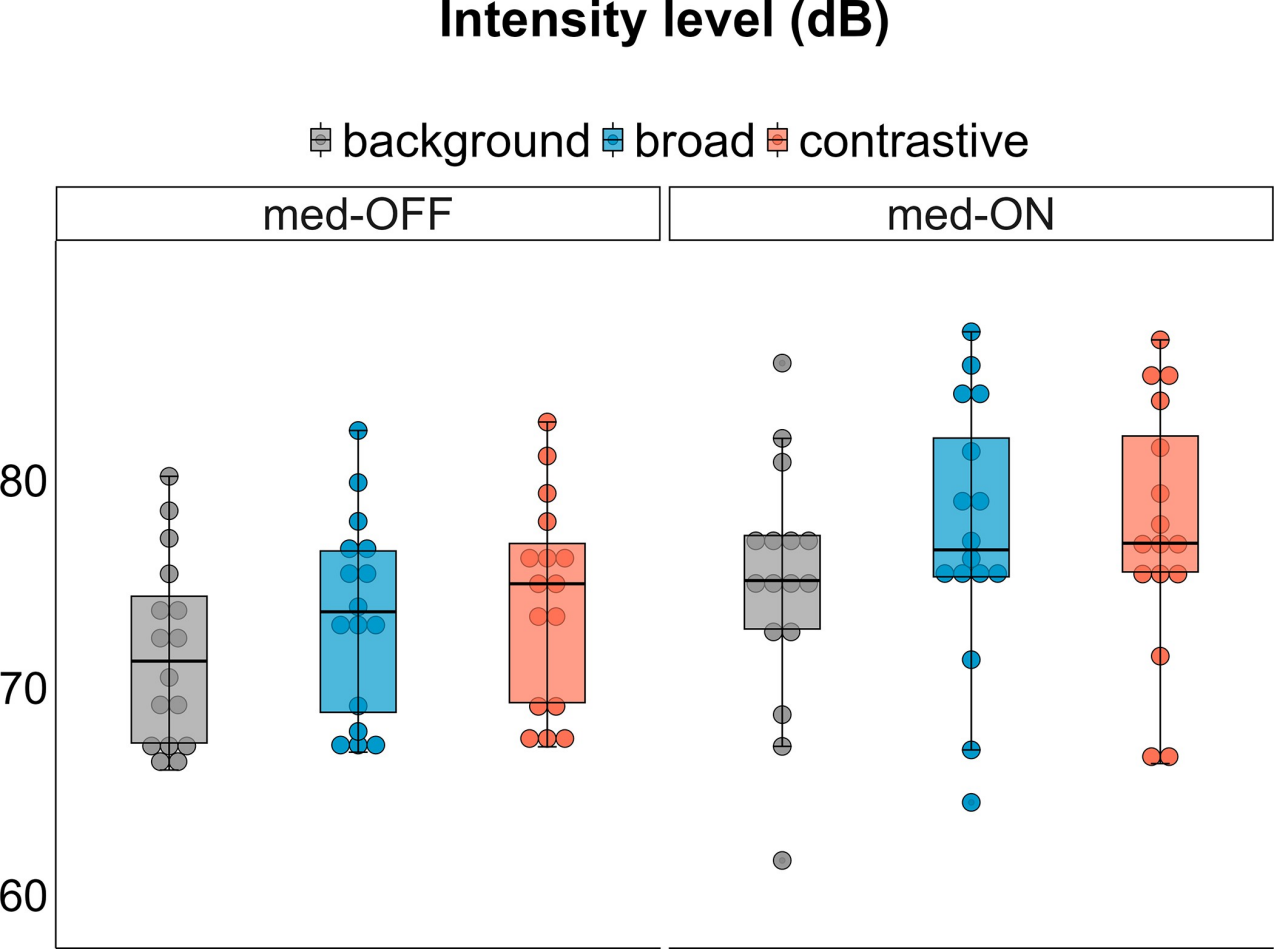
Averaged vocalic intensity of the V1 vowel per medication condition and focus category.

The statistical model does not reveal a significant interaction between condition x focus [X^2^(2) = 1.5755, p > .05], but shows main effects for both terms on intensity (condition: [X^2^(1) = 319.9, p < .001], focus: [X^2^(2) = 142.78, p < .001]). Post-hoc comparisons indicate that intensity values increase by 3.51 dB from OFF to ON condition (p < .001, Fig 3). Furthermore, intensity is increased for prominence production across (background vs. broad: mean difference = 2.081 dB, p < .001 | background vs. contrastive: mean difference = 2.584 dB, p < .001) but not within accentuation (broad vs. contrastive).

### Acoustic syllable duration

**Fig 4:**
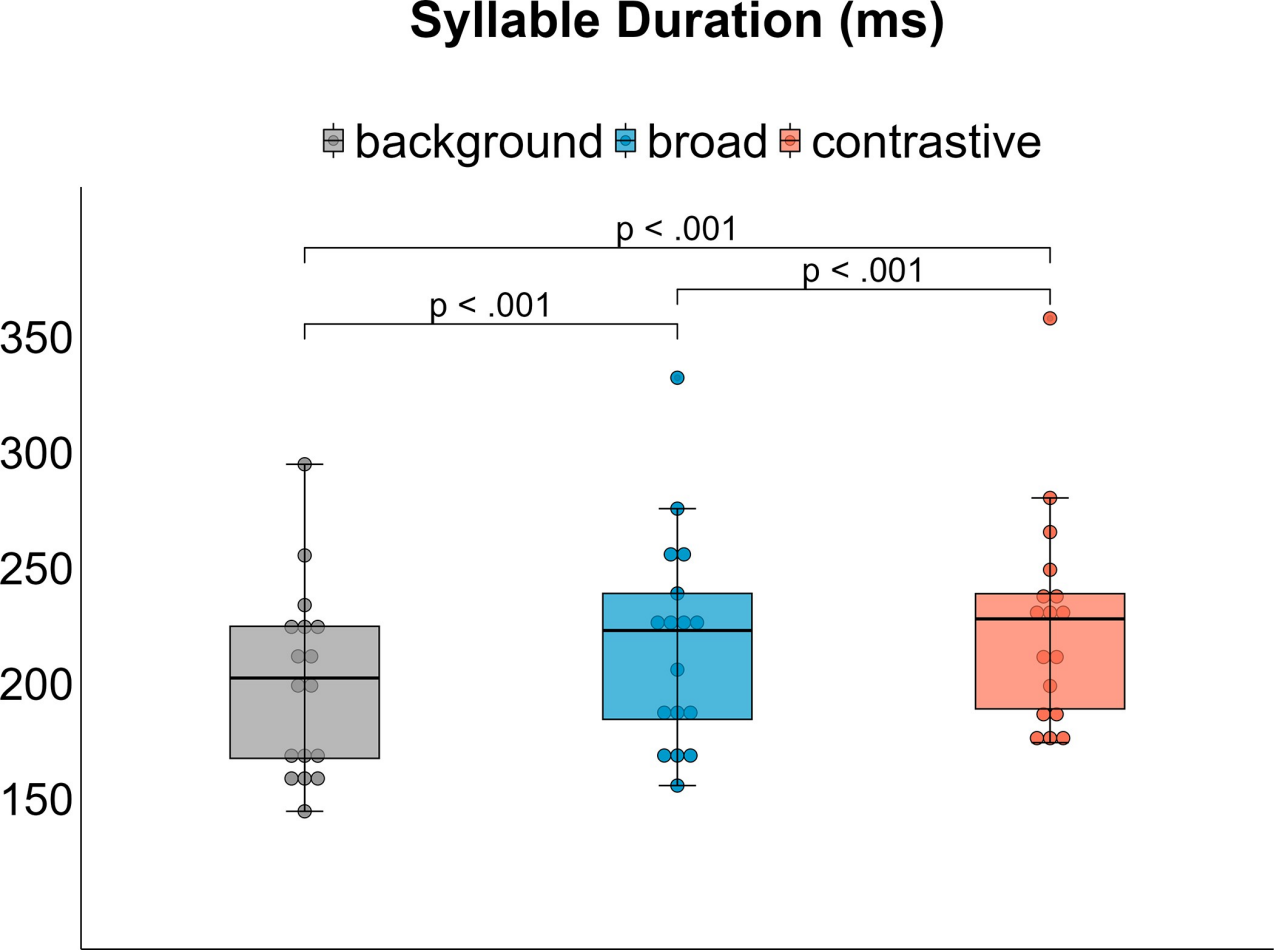
Averaged syllable duration per focus category averaged over medication conditions.

The statistical model neither reveals a significant interaction between condition x focus [X^2^(2) = 2.6273, p > .05] nor a main effect of condition on syllable duration [X^2^(1) = 1.036, p > .05]. However, there is a significant focus effect [X^2^(2) = 170.39, p < .001]. Post-hoc comparisons indicate that syllable durations increase for prominence production across (background vs. broad: mean difference = 25.53 ms, p < .001 | background vs. contrastive: mean difference = 17.06 ms, p < .001) and within accentuation broad vs. contrastive: mean difference = 8.46 ms, p < .001, Fig 4).

### Kinematics of oral articulators

The following section presents the spatio-temporal results of lower lip, tongue tip, and tongue body kinematics related to the target syllables. Means and standard deviations for the measures are displayed in Table 4.

**Table 4:** Means and standard deviations of kinematic parameters per medication condition and focus category. Durations are specified in ms, amplitudes in mm.

	**Lower Lip Duration**			**Lower Lip Amplitude**		
	background	broad	contrastive	background	broad	contrastive
OFF	104 (25)	103 (23)	107 (25)	4.1 (2.1)	4.6 (2.5)	4.7 (2.5)
ON	101 (24)	107 (29)	107 (31)	4.8 (2.3)	4.9 (2.6)	5.0 (2.8)
	**Tongue Tip Duration**			**Tongue Tip Amplitude**		
	background	broad	contrastive	background	broad	contrastive
OFF	92 (27)	98 (27)	99 (28)	4.1 (2.7)	4.5 (2.7)	4.4 (2.7)
ON	93 (23)	97 (30)	100 (30)	4.0 (2.4)	3.9 (2.6)	4.1 (2.5)
	**Tongue Body Duration**			**Tongue Body Amplitude**		
	background	broad	contrastive	background	broad	contrastive
OFF	202 (47)	216 (50)	218 (52)	7.3 (4.0)	7.8 (4.4)	7.9 (4.4)
ON	198 (43)	213 (50)	221 (57)	7.2 (4.2)	7.6 (4.5)	8.0 (4.5)

### Lower lip

**Fig 5:**
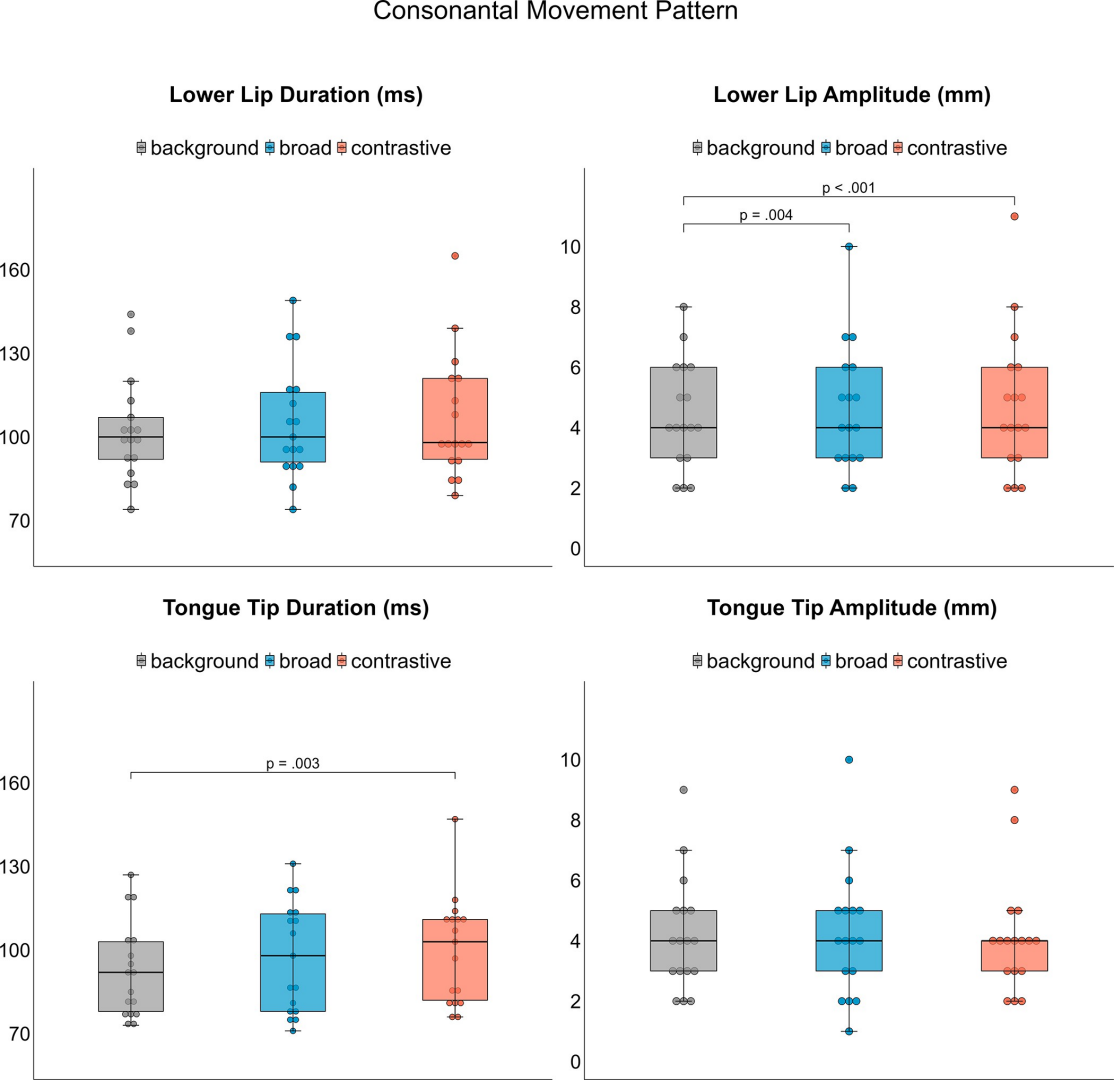
Articulatory results for consonantal lower lip and tongue tip movements per focus category averaged over medication condition.

No interaction [X^2^(2) = 1.5376, p > .05], no focus effect [X^2^(2) = 5.5359, p > .05], and no medication condition effect [X^2^(1) = 0.0502, p > .05] were found for lower lip movement duration (Fig 5 top left). For lower lip amplitudes, there was no interaction found [X^2^(2) = 2.2871, p > .05], but a medication condition effect [X^2^(1) = 17.083, p < .001] and effect of focus category was found [X^2^(2) = 16.429, p < .001]. Amplitudes are about 0.426 mm larger in ON condition compared to OFF condition. In addition, lower lip amplitudes increase across accentuation (background vs. broad: mean difference = 0.4044 mm, p = .004 | background vs. contrastive: mean difference = 0.4752 mm, p < .001) but not within accentuation (Fig 5 top right).

### Tongue tip

No interaction [X^2^(2) = 0.2364, p > .05] and no medication effect [X^2^(1) = 0.0184, p > .05] on tongue tip movement duration were found, but there was an effect of focus category [X^2^(2) = 10.956, p = .004]. Post-hoc comparisons indicate increased duration from background to contrastive focus (mean difference = 7.09 ms, p = .003, Fig 5 bottom left). While there was neither an interaction [X^2^(2) = 2.7048, p > .05] nor an effect of focus category [X^2^(2) = 3.4417, p > .05] found on tongue tip amplitudes, there is a significant main effect for medication condition [X^2^(2) = 6.374, p = .012]. Post-hoc comparisons reveal that movement amplitudes are about 0.289 mm smaller in ON condition compared to OFF condition.

### Tongue body

**Fig 6:**
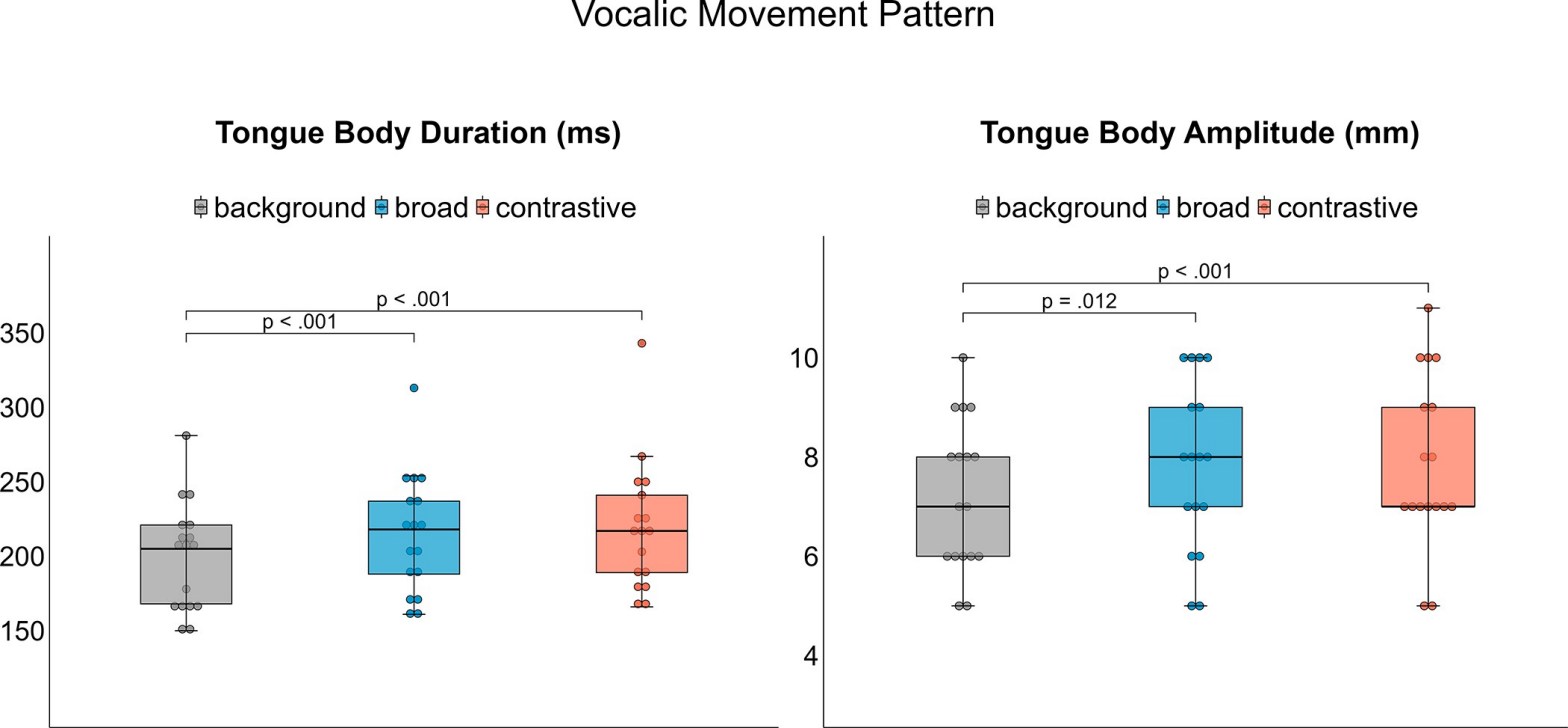
Articulatory results for vocalic tongue body movements per focus category averaged over medication condition.

No interaction [X^2^(2) = 2.3611, p > .05] or main effect of medication condition effect [X^2^(1) = 0.423, p > .05] were found for tongue body movement duration, but an effect of focus category exists [X^2^(2) = 68.182, p < .001]. Post-hoc comparisons reveal increased tongue body movement durations across accentuation (background vs. broad: mean difference = 14.92 ms, p < .001 | background vs. contrastive: mean difference = 19.67 ms, p < .001, Fig 6). While no interaction [X^2^(2) = 0.6176, p > .05] and no main effect of medication condition [X^2^(1) = 0.4024, p > .05] was found on tongue body amplitude, there was a significant effect of focus category [X^2^(2) = 16.108, p < .001]. Post-hoc comparisons reveal changes in amplitude across accentuation (background vs. broad: mean difference = 0.511 mm, p = .012 | background vs. contrastive: mean difference = 0.693 mm, p < .001) but not within accentuation (Fig 6).

## Discussion

In what follows, we discuss our findings with respect to the ability of PwPD to modulate the speech system to encode prominence. In line with findings from healthy speakers of German, intensity and articulation were predominantly used to distinguish between accented and unaccented syllables (out-of-focus versus focus), while F0 and syllable duration were sensitive to differentiate focus types (broad focus versus contrastive focus). This was indeed the case regardless of motor condition, and might be due to (a) a mild state of dysarthria in the examined speakers and (b) a goal-directed effect in the speech task used in our study. We will further discuss levodopa effects on speech performances, revealing main effects of medication on the respiratory-phonatory system as well as a more agile lip and tongue system on the kinematic dimension under medication. We conclude with discussing the limitation of the present study.

### Prominence marking

Prosodic modulations were controlled with this study design to analyze whether PwPD are capable of adjusting speech parameters according to the varying demands within communicative contexts. The investigation of prominence marking strategies in particular was considered in order to determine if PwPD differentiate between three different focus categories by adjusting speech parameters on the continuum from hypo- to hyper-articulated speech [16].

The results suggest that the ability to encode information structure is maintained in predominantly mild dysarthric PwPD, regardless of motor condition. Already two previous studies proposed that the linguistic function of prosodic prominence is preserved in PwPD [20, 21]. While pitch modulations are used to differentiate across accentuation (background vs. broad focus, background vs. contrastive focus) and within accentuation (broad vs. contrastive focus), intensity values are adjusted only across accentuation. This is in line with recent research on prominence marking in neurotypical speech in German, stating that the differentiation between focus types with accented target words is generally more subtle than those across accentuation [3]. With respect to the relative importance of phonetic cues, Roessig et al. described intensity modulations as relevant only across accentuation, while pitch changes are the strongest correlate of prosodic prominence within and across accentuation [3].

In our dataset, we further found modulations on syllable durations on the segmental level to be relevant for focus expression in PwPD. Acoustic syllable durations were modulated across and within accentuation to produce different degrees of prominence. When looking at the sub-syllabic level of consonants and vowel production, we found spatio-temporal modification of articulatory features in speech kinematics. Movement durations of the tongue tip and the tongue body increased across accentuation. Since longer durations are perceptually associated with an increase in sonority, PwPD make use of the sonority expansion strategy [65, 66] to highlight the accented target word. Regarding the spatial domain, more extreme tongue positions were observed under prominence which were reflected in larger tongue body amplitudes indicating an increase in vowel space. Thus, PwPD also make use of the hyper-articulation strategy with the aim of enhancing prosodic contrasts to encode focus structure [9–11]. However, vocalic features were only adjusted across accentuation and do not express different degrees of prosodic prominence when comparing accented target words in broad and contrastive focus. This pattern is in line with recent research reporting that spatial articulatory positions are already peripheral in broad focus condition and might not be further expanded in contrastive focus condition [3]. Although the vowel is the main area of prominence marking, lower lip amplitude was additionally adjusted across accentuation in this dataset. An increase in lip aperture allows for an increase of acoustic energy radiating from the mouth. This is in line to increased lip opening that was observed during vowel production in neurotypical speech patterns of German before [11, 67, 68].

To sum up, PwPD with mild dysarthria adjust prosodic speech parameter similar to patterns that were previously observed in neurotypical speech by modulating acoustic durations and pitch ranges across and within accentuation, and spatial features and intensity across accentuation [3]. In other words, PwPD adapt to communicative demands, and maintain prominence relations by modulating the respective prosodic parameters.

### Levodopa effect

Only PwPD that responded sufficiently to the supratherapeutic levodopa dosage were included in the analysis. Motor functions which were assessed with the UPDRS III improved on average by 55% under levodopa. With regards to speech functions, only respiratory-phonatory features of speech, such as intensity [30] and pitch range [22] were responsive to levodopa, while acoustic syllable duration was not [30, 32, 34, 38]. This is in line with previous assumptions proposing that respiratory-phonatory features of speech are responsive to levodopa, while articulatory features are not [69, 70].

Improved respiratory-phonatory functions under levodopa were reflected in louder speech of PwPD in medication-ON condition. While the overall pitch range did not differ between medication-OFF and medication-ON condition, pitch range in broad focus production increased under levodopa indicating improved phonatory control. The results are contradictory to a study that stated that prosodic features might not be controlled in dopamine-dependent brain circuits, as the functional connectivity within brain networks that are related to prosody control did not change due to levodopa intake [71]. However, our study supports the idea that phonatory markers of prosodic prominence improve under levodopa. However, our results are in discrepancy to some previous studies. While the control of pitch and intensity significantly differed in our study, it did not change under levodopa in others [30, 33–37]. Reasons for that might lie in the variable nature of different speech tasks that were used throughout the studies as well as related measures which were either calculated global across entire sentences or local within single syllables. Our study focused on local changes which might be more sensitive than detecting global changes of averaged values across entire sentences or paragraphs.

Additionally, our study was able to detect changes on the kinematic level. Larger lower lip movements and smaller tongue tip movements were observed under levodopa, reflecting a more agile speech system. While previous studies have reported spatial and temporal changes [42–44], this study supports the results of spatial changes of consonantal movements. On the other hand, levodopa intake did not affect tongue body movements. Lacking effects on the tongue body could be explained by the high degree of articulatory freedom of vocalic movements [72, 73], as there are many more ways to achieve a vocalic target than a consonantal target. Furthermore, vocalic movements are by nature slower than consonantal movements. The results highlight the fact, that the consonantal system is differently affected by levodopa intake compared to the vocalic system. With regards to recent studies, one could also assume that the speech impairment severity was too mild and the prosodic system not too severely impaired so that levodopa could lead to a great improvement [45, 46].

### The role of goal-directed behavior in lab speech tasks

As PwPD were able to produce prosodic prominence patterns regardless of motor condition, we want to explain these results by discussing the difference between goal-directed vs. habitual behavior which is controlled in different loops of the basal ganglia. Habitual behavior is known to be highly impaired in PD whereas goal-directed behavior can be preserved over a longer period of the disease. According to the model of Redgrave et al., goal-directed behavior involves frontal cortical areas connected via loops with the associative basal ganglia (which are still functional in PD), whereas habitual behavior involves the sensorimotor cortex connected to the sensorimotor basal ganglia structures (which are dysfunctional in PD) [74]. Further and in contrast to the associate loop, the dysfunctional sensorimotor loop can be modulated by dopamine thus leading to improvement of (habitual) motor functions.

Brief digression: Physicians treating PwPD often observe goal-directed behavior in daily clinical practice. Caregivers of PwPD often report much better motor performance during the neurological examination compared to everyday performance, e.g. at home. This effect is often pronounced in gait, similar to speech, another highly automated (habitual) motor function. For example, freezing of gait, i.e. ineffective stepping despite the intention to walk [75], occurs less frequently in research settings compared to everyday life [76].

Since the sensorimotor pathways are in particular impaired in PD, goal-directed behaviors controlled via associative pathways can still be performed. Fulfilling specific speech tasks in a lab can be compared to goal-directed behavior. The questions-answer pairs used in our lab speech task to elicit focus structure might have led to stronger modulations in the speaker’s phonetic space when being compared with spontaneous speech of the same speaker. In this regard, our study design per se might provoke a bias to increase the phonetic cost for packaging linguistic information. The same might have been applied in an even stronger way for previous studies in which the participant was asked to explicitly highlight certain key words. This could explain why PwPD were able to fulfill the prominence task equally well as compared to neurotypical speakers as it has been shown before [58]. With respect the model of Redgrave et al. [74], goal-directed behavior for speech tasks are controlled in a motor network that works independent of dopamine. This explains why prominence marking strategies did not differ between motor conditions in our study.

However, the results obtained from our study cannot be directly transferred to everyday speech, as the experimental set-up might have prompted externally cued speech performance. Cueing has been reported for other PD symptoms, such as gait initiation, as a beneficial strategy to complete tasks [77, 78]. Therefore, cueing in our set-up might have caused PwPD to overperform in the prominence task, making their performance comparable to that of neurotypical speakers. In the future, speech performance should specifically be compared between habitual and goal-directed settings or between real-life conversation and lab-based speech.

### Limitations

The gender distribution is unbalanced and the disease duration varies across participants. However, the gender distribution is comparable to many other studies, as this illustrates the regular prevalence that men are more frequently affected than women. Further, the variation in the disease duration makes it possible to summarize the results more generally and not just at specific stages of the disease, such as early, advanced or late. In addition, the sample size is limited in this study, potentially restricting the generalizability of the findings. However, the sample size is larger compared to other kinematic studies investigating speech performance of PwPD. Further, administering a supramaximal dose of levodopa does not reflect the usual medication effect and carries potential risks of hyperkinesia. In the case of hyperkinesia, hyperactivation could have also influenced the PwPDs’ speech performance.

Despite these limitations, this study contributes to expanding our understanding of the effects of levodopa on the speech system, particularly on the kinematic level. It is crucial to consider these constraints when interpreting the results and to conduct further research to obtain a more comprehensive view of the topic.

## Conclusion

The linguistic function of prosody seems preserved in predominantly mild dysarthric PwPD independent of the medication state. Therefore, PwPD with mild to moderate dysarthria are able to adapt their speech system to communicative demands to signal prosodic functions relevant for social interactions. However, levodopa had beneficial effects on phonatory-respiratory speech features, such as loudness and pitch, but did not affect articulatory features of vowel production in this cohort.
